# ‘Adrift in a sea of just absolute unknowableness’: A multimethod qualitative study exploring patient, carer and healthcare professional experiences of communicating about future uncertainty in multimorbidity

**DOI:** 10.1177/02692163251393586

**Published:** 2025-12-01

**Authors:** Rosanna Fennessy, Anna Spathis, Stephen Barclay, Simon Noah Etkind

**Affiliations:** 1 Primary Care Unit, Department of Public Health and Primary Care, University of Cambridge, UK; 2 Cambridge University Hospitals NHS Foundation Trust, UK

**Keywords:** uncertainty, communication, multimorbidity, healthcare planning, older adults

## Abstract

**Background:**

Uncertainty about the future is commonly experienced by older adults with advanced multimorbidity, impacting multiple life domains and frequently causing anxiety. Communicating about future uncertainty affects patient satisfaction, quality of decision-making and opportunities for future planning. Patient, carer and healthcare professional experiences of future uncertainty communication have been little studied to date.

**Aim:**

In the context of older adults with advanced multimorbidity, to explore patient, carer and healthcare professional experiences and preferences for communication of uncertainty about their future.

**Design:**

Multi-method qualitative research utilising in-depth interviews and focus groups with reflexive thematic analysis.

**Setting/participants:**

Older adults with advanced multimorbidity (*n* = 15), their nominated informal carers (*n* = 3), and community and inpatient healthcare professionals working with this population (*n* = 17).

**Results:**

Participants’ experiences of uncertainty communication were influenced by their acceptance or avoidance of addressing future unknowns. Patients and their informal carers expressed a range of information needs: they prioritised open and honest dialogue with healthcare professionals, tailored to their unique circumstances and supported by reassurance and proactive plans, even when medical options were limited. Healthcare professionals acknowledged the importance of addressing uncertainty: they expressed a lack of experience and confidence and feared upsetting patients or losing their trust. As a result, opportunities for timely and personalised communications may be missed.

**Conclusions:**

To support patients and carers navigate the inherent uncertainties of advanced multimorbidity, healthcare professionals would benefit from tailored communication training designed to increase their experience of undertaking honest and open dialogue about future uncertainties, informed by patients’ individual context and preferences.

**What is already known about this topic?**

- Multimorbidity affects increasing numbers of older people worldwide: the complexity of managing multiple illnesses creates future uncertainty for patients, carers and healthcare professionals.
- Uncertainty impacts physical, psychological, social and existential domains and is often distressing for patients and carers.
- The ways in which healthcare professionals communicate about future uncertainty impacts patient satisfaction and decision-making, including treatment choices.

**What this paper adds**

- Patients, carers and healthcare professionals have a mixture of acceptance and avoidance when considering future uncertainty, which influences the way communication is undertaken and experienced.
- Mismatches in communication preferences may lead to missed opportunities for conversations about the future that would reduce patient anxiety.
- Patients and informal carers perceive communication with healthcare professionals about future uncertainty to be suboptimal when it does not address their particular circumstances and priorities, further intensifying their distress from uncertainty.
- Healthcare professionals, particularly those with less experience, may struggle to distinguish between solvable uncertainties due to lack of clinical information and inherently unknowable uncertainties.

**Implications for practice, theory or policy**

- Open expression of uncertainty in the context of a trusting relationship is welcomed by most patients and carers: healthcare professionals need to honestly communicate uncertainty rather than avoiding it.
- Expressing uncertainty alone is insufficient: communication needs to take account of patient preferences within a framework of continued support that offers options for action.
- Healthcare professionals caring for patients with advanced multimorbidity would benefit from education focused on creating a personalised approach to uncertainty communication.

## Introduction

Uncertainty is experienced by patients, their informal carers and healthcare professionals, and may relate to diagnosis, management, and the future.^1,2^ Navigating these uncertainties is a core part of healthcare professionals’ work.^3–5^ Uncertainty impacts multiple life domains and can be a source of great distress: it can adversely affect appropriate decision-making if not effectively addressed.^2,6,7^ At its worst people may experience overwhelming ‘Total Uncertainty’ defined as uncertainty across multiple domains of experience including physical, psychological, practical, social and temporal.^2,8^ Uncertainty can be exacerbated for patients and carers by the sheer volume (and often inconsistency) of medical information in the public domain, while healthcare professionals can face a dilemma between knowing whether a specific uncertainty is due to personal lack of knowledge, or whether the issue of uncertainty is beyond current science to resolve.^3,5^

Key drivers of uncertainty are complexity and unpredictable future illness trajectories, both of which are commonly experienced in older age and by those with multiple serious illnesses (advanced multimorbidity).^9,10^ Terminology in this area is inconsistent, with no agreed definitions;^11^ here we define the term advanced multimorbidity as the co-existence of at least two serious or life-limiting illnesses such as cancer or organ failure. Numbers of individuals with advanced multimorbidity are predicted to rise globally, and to account for over 50% of deaths by 2040 in high income countries.^12,13^

Uncertainty about the future is prevalent in multimorbidity, with inter-individual variation and unpredictability in the trajectories of multiple conditions making prognostication particularly challenging.^6,14–17^ In health care systems primarily designed for single conditions, patients often experience fragmented care across different specialist teams that creates further dimensions of uncertainty regarding possible outcomes.^2,9,18^ Given the irreducible element of future uncertainty for many patients, care needs to be delivered in its presence, with practitioners working with patients and carers to achieve a position of ‘safe uncertainty’ where ambiguity is reduced by addressing known unknowns in partnership.^19,20^

Skilled communication improves patient-centred outcomes, patient satisfaction and trust in healthcare professionals and services.^21–25^ Patient-centred communication can improve care co-ordination and provide a sense of control in the presence of unpredictability.^6,26,27^ While uncertainty communication is a recognised research priority,^28–30^ studies have largely focused on prognostic communication, often using simulated patient interactions or vignettes.^23,31–34^ Little research has addressed patient, carer and healthcare professional perceptions and experience of uncertainty communication, particularly in multimorbidity, where the future is often very unpredictable. We aimed to explore patient, carer and healthcare professional experiences and preferences for communicating about future uncertainty in the context of older adults with advanced multimorbidity.

## Methods

### Study design

To explore experiences of uncertainty communication across different contexts, we undertook a multi-method, multi-perspective study using in-depth interviews with patients and carers and focus groups with healthcare professionals. Data were collected as part of a larger co-design study which develops recommendations for communication of uncertainty in advanced multimorbidity, which is reported in detail elsewhere.^35^ Reporting follows the Standards for Reporting Qualitative Research (SRQR).^36^ PPI is reported according to the GRIPP2 checklist.^37^

### Setting and population

Eligible participants were older adults with advanced multimorbidity, their informal carers and healthcare professionals who have a role in communicating uncertainty with this population. To capture heterogeneity in the advanced multimorbidity population and variation of views about the future course of illness, we aimed to recruit patients with a similar degree of illness severity but who were accessing different services with a different focus of care. To achieve this, we recruited from outpatient and community palliative care teams at two hospices and from an inpatient rehabilitation unit. Eligible healthcare professionals were from multiple settings where communicating about future care with multimorbid older adults is commonplace, including palliative care, rehabilitation and geriatrics.

### Sample

Patient participants were purposively sampled to be aged >65 with advanced multimorbidity, defined as the presence of at least two serious health conditions (operationalised as those included in a modified version of the Murtagh et al. estimate of population palliative care need).^38^ (see Supplemental File 1).

Participants were excluded if they lacked cognitive or physical ability to participate in an interview. Sampling aimed for maximum variation across settings, diagnoses and healthcare professional occupations. Data collection continued alongside analysis, until the research team considered we had sufficient information power to answer the focused research aim. For patient participants, this involved exploring experiences of communicating future uncertainty in the population of interest, rather than communication in more general senses. It was not feasible to include all potentially relevant diagnoses, but instead we focused on depth of exploration and identifying common threads of experience when assessing information power.^39^

### Recruitment

Potential patient participants were approached by clinical staff with whom they had an existing relationship. They explained the study, gave them an information sheet and if verbal consent was given, they were contacted by the research team (RF or SE). Informal carers were nominated by consenting patients. Healthcare professionals were invited via clinical networks linked to the settings in which we undertook patient recruitment. All participants provided written consent.

### Data collection

Due to the sensitive nature of the topic and to facilitate participation, we undertook in-depth individual interviews either in patients’ homes or the in-patient rehabilitation setting, between May 2023 and January 2024. Carers who consented were offered the option of separate or joint interviews with patients. We were mindful of the trade-off between potentially gathering more insights from separate interviews but possibly greater acceptability of joint interviews to maximise participation. We conducted focus groups with healthcare professionals from a range of professional backgrounds to allow for generation of deeper insights through discussion. We used topic guides covering (a) experiences of uncertainty in multimorbidity and (b) communication about future uncertainty (see Supplemental File 2). These guides were developed from the existing literature on uncertainty and refined by patient and public involvement (PPI) contributors. Interviews and focus groups were audio recorded, transcribed and anonymised prior to analysis.

### Data analysis

#### Analysis approach and reflexivity

Analysis followed a reflexive thematic analysis approach to identify patterns of meaning across the dataset.^40,41^ We adopted a constructivist perspective which acknowledges that each participant’s unique perspective on future uncertainty communication is created by their interactions with others.^42^ Data were analysed inductively by a palliative care physician-researcher (SE) and a non-clinical research psychologist (RF). Both researchers completed contemporaneous written reflections and reflexive diaries throughout analysis. We undertook semantic coding,^40^ focused explicitly on participants’ descriptions of their experience and views of future uncertainty communication, rather than interpreting latent meanings within their dialogue. Discussion and reflection with the research team and our patient and public advisory group with lived experience, continued throughout analysis.

#### Analysis steps

- Data familiarisation included reading the transcripts twice.
- The same three patient and carer transcripts and one focus group transcript were coded independently by SE and RF, adopting a data driven approach to create initial codes linked to the research aim. These were compared to identify broad areas of agreement and disagreement resolved by discussion to ensure a co-ordinated approach.
- The remaining patient and carer transcripts were coded by RF, and the healthcare professional transcripts by SE. Both researchers generated initial themes.
- Initial findings from all transcripts were jointly mapped by SE and RF, exploring similarities and differences in researcher coding and experiences within and between participant groups. Areas of consensus and discrepancy were collaboratively addressed with input from the wider study team.
- Themes were presented to the study team and PPI members to further refine the analysis. PPI involvement ensured that themes reflected different patient and carer experience.

### Ethics

The study was approved by the Oxford C Research Ethics Committee in February 2023, ref: 23/SC/0024.

## Results

We interviewed 15 patients with advanced multimorbidity. Three patients identified carers who opted for joint interviews (see Table 1). Three focus groups (two face to face, one online) comprised 17 healthcare professionals across a range of roles (see Table 2).

**Table 1.** Patient and informal carer participant details.

Patients	Details (*n*)
Gender	M (7), F (8)
Age	Median 81 years, range 70–89 years
Ethnicity	White (15)
Recruitment setting	Palliative care^a^ (10) In-patient rehabilitation (5)
Number of patients with each eligible diagnosis^b^	Cancer (7) Cardiovascular disease (11) Respiratory disease (10) Renal disease (2) Liver disease (1) Dementia/neurological disease (3) Musculoskeletal disease (8)
Clinical frailty score	4 20% 5 40% 6 40%
Informal carers	Details (*n*)
Gender Ethnicity Age	M (1), F (2) White (3) Median 76 years, range 65–77 years

a Palliative care settings were outpatient clinics and ‘living well service’ /day hospice at two hospices, designed for patients with life limiting illnesses but with a prognosis of months to years.

b Health conditions were patient reported. Each patient had at least two eligible diagnoses.

**Table 2.** Healthcare professional participant details.

Healthcare professionals	Details (*n*)
Gender	M (5) F (12)
Age	Median 44 years, range 20–66 years
Ethnicity	Asian (5), mixed (1), white (11)
Occupational setting	Palliative care (6) Rehabilitation (7) Geriatrics (4)
Healthcare professional role	Palliative care physician (2) Geriatrics physician (4) Nurse, nurse specialist, or healthcare assistant (5) Allied health professionals (5) Chaplain (1)
Years experience	< 1(1) 1–4 (1) 5–9 (4) 10+ (11)

Five inter-connected themes mediated patients’, carers’ and healthcare professionals’ experiences of future uncertainty communication:

- Perceptions about the nature of uncertainty,
- Responses to uncertainty,
- Personalised care relationships,
- Healthcare professional experience and confidence, and
- Perceived degree of honesty and openness.

All themes were recognised across patient, carer and healthcare professional participants, though some were more prominent for particular group(s).

### Perceptions about the nature of uncertainty

Experiences of communicating future uncertainty were shaped by perceptions of its nature. All participants recognised multiple types of uncertainty, but we noted differences in emphasis. For healthcare professionals, future uncertainty was seen as a core concept in their work, affecting many aspects of their everyday practice and communication, whereas uncertainty was often experienced more implicitly by patients and carers.

When patients and carers did consider future uncertainty, they were more likely to focus on the day-to-day uncertainties associated with having multiple unpredictable illnesses, such as impacts on family or participation in household activities. However, some patients and carers did acknowledge broader awareness that the future is inevitably uncertain and unknowable.

*Yes, yes, and adapt to it [future uncertainty] and not be afraid of it. Because I know it’s going to happen, but the only thing I worry about is my family seeing me. You know, that sort of thing, that’s the only thing that worries me. How will they feel, you know? [Interview – patient with cancer and musculoskeletal disease]*

In contrast, healthcare professionals recognised a tendency to focus on a narrower biomedical definition of uncertainty, often centring around prognosis. Focusing too much on prognosis was recognised as potentially problematic.

*You’re not identifying any of the uncertainties that the patient’s really worried about, and so the focus of the consultation is very biomedical and ignores the uncertainties altogether. [Focus group – healthcare professional, palliative care]*

All participants recognised that health system factors such as access to care and continuity across multiple providers, created additional uncertainties. Many patients commented on the feeling that their care was disjointed, and that uncertainty was created by insufficient communication between their different care teams.

*You go from one to the other and get nowhere because consultants these days, they do their cardiology, someone else does rheumatology, someone else does this and they don’t get together and look at the whole picture whereas, that is very frustrating because one won’t be in contact with the other, it doesn’t make sense but sometimes we were up against that. [Interview – patient with cardiovascular and respiratory disease]*

### Responses to uncertainty

When faced with future uncertainty, participants’ differing responses affected communication experiences and preferences. Emotional reactions included frustration, anxiety and feeling overwhelmed or ‘paralysed’ by future uncertainty; these reactions could lead to acceptance, avoidance or seeking reassurance.

#### Acceptance

Some participants perceived uncertainty as an inevitable aspect of life with multimorbidity and were more comfortable with acknowledging an uncertain future, although this could be a barrier to discussion or planning ahead.

*Yeah, we lived for the moment, why worry about the future, we don’t know what the future is. You could go out and fall over and break your neck. You know, that’s our attitude to life and some people say blasé but we don’t. . . Why waste time thinking about things that you’ve got no control over because there’s nothing you can do about it. [Interview – patient with renal and liver disease]*

Healthcare professionals acknowledged that in the presence of multimorbidity, focusing on reducing uncertainty could be a barrier to timely care and that some uncertainty had to be accepted. Some healthcare professionals found it hard to accept uncertainty, which could also impede open discussions.

*Because I think if you can’t accept uncertainty and you’re forever trying to make a diagnosis or precisely define what will happen and you can’t, that’s a barrier to just getting on and doing the best you can, which is, yeah, that’s what sometimes we need to do. And that’s a challenge. We’re taught and trained to be precision perfectionist types. [Focus group – healthcare professional, geriatrics]*

#### Avoidance

For some participants, thinking about or discussing an uncertain future was to be avoided, often in favour of a focus on day-to-day living or other priorities which aided a sense of control, or enabled a positive focus which fostered hope.

*I can’t think about that now, I need to leave that till another day, and I suppose a lot of the uncertainty, that’s what I’d, you know, how I deal with it, I can’t think about it now, it’s too hard, it comes in the too hard category. [Interview – carer of patient with cancer, cardiovascular and respiratory disease]*

However, avoiding uncertainty could also inhibit discussion, resulting in ‘decision paralysis’.

*At its worst it’s almost paralysing. A chap on the inpatient unit at the moment who is, he and his wife are just so hyper-anxious and I think almost all of that comes from the sense of loss of control which is strongly associated with the uncertainty and a conversation we had last week which was the first time that he’d ever really opened up about being, felt able to talk about any of this. [Focus group – healthcare professional, palliative care]*

Some healthcare professionals avoided addressing uncertainty, reporting that communication wasn’t comfortable until uncertainty was removed; they feared upsetting the patient or introducing further uncertainty.

*You’re actually introducing uncertainty, something that might not have been an issue for them before and then mention something and they think “oh gosh, that’s not something I’ve thought of”. [Focus group – healthcare professional, geriatrics]*

#### Seeking reassurance

Both acceptance and avoidance could inhibit communication of uncertainty, however, some patients approached uncertainty directly, turning to others to help address it. This included wanting healthcare professionals to give proactive advice and suggestions for action, despite potential unknowns.

*I don’t mind them saying they’re not sure. I do mind them saying “we don’t know, goodbye.” If they say they’re not sure, but we’ll have a look at this, fine, you know. [Interview – patient with cardiovascular and respiratory disease]*

At times, the inherent complexity of multimorbidity meant that some healthcare professionals found certainty around their decision-making challenging, which could leave their patients’ need for reassurance unfulfilled.

*When there’s uncertainty on top of uncertainty then sometimes it can be, I find, I’m less confident that I’ve made, you know, the right decision because there’s so many kind of variables at play. [Focus group – healthcare professional, geriatrics]*

### Personalised care relationships

Patients and carers placed great value on a strong care relationship with a practitioner who knew them as individuals and their particular wishes and spectrum of medical problems. Where patients felt their individual needs and wishes were listened to, particularly in relation to the unique nature of their multiple conditions, this supported open communication and facilitated a two-way dialogue.

*. . . this doctor here she’s lovely, she’ll tell me the truth because she knows me, this is the thing, think once a doctor or surgeon or whatever gets to know you, and it’s, continuity in care has gone, but I like to see the same people and if you do you’re lucky and then they get to know you and they know just what you want. [Interview – patient with cardiovascular disease, liver disease and cancer]*

Patients and carers reported that if an individual relationship wasn’t established, communication could become generic, leaving them feeling unheard and reluctant to share their concerns. Without time to develop rapport, meaningful dialogue was inhibited and uncertainties left uncommunicated.

*When you go in, it’s just, “How are you? Are you okay? Okay. See you in six months, bye”, you know, that sort of thing. But you don’t feel like you can let them know really what’s happening. You come out and you think what was the point of that. [Interview – patient with cardiovascular and respiratory disease]*

Some healthcare professionals highlighted the importance of focusing on the individual circumstances and preferences of patients, beyond their physical health. Exploring patients’ wider needs enabled healthcare professionals to support patients with the challenges of facing an uncertain future and prevented patients losing trust in them and the healthcare system.

*The other word is the uniqueness because although you’ve got the tools and you could have a pathway, every situation as we said could be physical, could be emotional, could be spiritual, and even that individual’s very unique. So there’s no set path is there? There’s no set rule. We’re led by the patient. [Focus group – healthcare professional, palliative care]*

### Healthcare professional experience and confidence

In part because of the lack of an evidence base, healthcare professionals reported a tension between future uncertainty being the result of something that can’t be known or was due to their lack of knowledge or experience. This was a particular problem for less experienced participants in our sample and led to insecurities when seeking to give patients definitive answers.

*I also think first the distinction between sharing uncertainty which is unknowable by anybody, so it’s at the limit of medical knowledge for whatever reason . . . versus uncertainty that’s due to your own lack of clinical expertise or your own lack of knowledge in a particular area. [Focus group – healthcare professional, geriatrics]*

Patients and carers expected healthcare professionals to be skilled in communicating future uncertainty and their ability to do this was integral to maintaining trust. Healthcare professionals were aware of this expectation and acknowledged the sometimes difficult balance between communicating uncertainty and maintaining trust.

*It’s communicating it in a way that still makes them feel like they’ve got confidence and trust in you to look after their relative or to look after them, whilst understanding that you don’t have all the answers and that’s a difficult balance sometimes to tread. [Focus group – healthcare professional, geriatrics]*

Whilst all healthcare professional participants frequently cared for older people with multimorbidity, staff from the rehabilitation unit felt that because of their focus on a population with potential to improve functionally, they were less prepared to address wider uncertainties about overall prognosis and end of life care.

*I suppose also because we’re a rehabilitation unit, we’re used to having just the positive discussions, whereas I suppose because we’re not a palliative care unit or a hospice we haven’t really had training in having those chats with people. [Focus group – healthcare professional, rehabilitation]*

Experienced healthcare professionals, mainly consultants and clinical nurse specialists, commented that it requires bravery to acknowledge and address their own uncertainties. This was particularly the case for doctors, who described the ‘culture’ of medicine as focusing on narrowing uncertainty, and their training didn’t necessarily support them to balance the need to reduce uncertainty, with the reality of its irreducible nature in multimorbid patients.

*And so I think some of the challenge is to, you know, is cultural to say “I have to learn to be able to deal with a wider uncertainty than I would like”, and I think that’s part of . . . what medical training should help you to do, as well as making you better at narrowing uncertainty, there are these kind of two poles that the more senior you are the more you want to be able to handle uncertainty and the better you want to be technically at reducing it. [Focus group – healthcare professional, geriatrics]*

Healthcare professional discomfort with addressing uncertainty and fears of losing patient trust was at odds with the views of patients and carers in our study who did not necessarily expect healthcare professionals to have all the answers. This could result in opportunities for dialogue to be missed.

*I think just be straightforward and say you know, ‘’I’m sorry I can’t, we don’t know what’s going to happen’, it’s a case of wait and see”*.

[INTERVIEWER]. And . . . if then someone said that to you, how would you feel?

*“Well I’d take it as that was the answer, nobody knows what’s going to happen.” [Interview – patient with cardiovascular and musculoskeletal disease]*

### Perceived degree of honesty and openness

Honesty and openness were seen as crucial to uncertainty communication. Where an open conversation was grounded in a personalised care relationship, with experienced and confident healthcare professionals, patients and carers were able to express their concerns and wishes, and healthcare professionals were able to more comfortably articulate where things are unknown.

*On that point of being honest I think it’s a lot easier to be honest if you’ve got the pre-existing relationship. If in the first five minutes of your consultation you’re kind of just going and saying “well we don’t really know”, then that’s really hard. [Focus group – healthcare professional, palliative care]*

Honesty and openness also served to allay additional patient anxiety, and could facilitate decision making and preparation for an unknown future.

*If they tell you then at least you can prepare and get your mind around it, and they can give you help with the decision, if you like. But if they don’t tell you, and they’ve got to hum and haw about everything all the time, then you’re going to be anxious, wondering why they’re doing certain things. [Interview – patient with respiratory and cardiovascular disease]*

Many patients and carers understood a great deal about the unpredictability of their health conditions and saw avoidance of addressing future uncertainty honestly as unwelcome. Openness was important to patients as it allowed them to understand that despite no definitive answers, healthcare professionals were still actively engaged in their care. Some patients and carers reported that they did not always experience openness, but rather a lack of clear communication around accessing support in complex healthcare systems, or discussion of future possibilities.

*They can’t say, “oh yeah, in 3 months’ time you’ll be this, that or the other”, but I don’t know, it’s just the way they explain things sometimes and the time they don’t have to do it but if. . . If they said, “oh yeah, well no, we don’t know what is in the future, but we will do our best and things”, but it doesn’t really happen like that, does it. [Interview – patient with cardiovascular and respiratory disease]*

Healthcare professionals acknowledged patients’ needs for openness and honesty but were conscious that they did not always have the full picture. As a result, their limited information could be perceived negatively by patients and carers.

*I think also if you’re looking after one patient with one illness and kind of go down a sort of basic trajectory but when you’ve got several different things you can’t be as certain. . .in your answers because you can’t predict or answer categorically when there’s lots of different things going on. [Focus group – healthcare professional, palliative care]*

## Discussion

This multimethod qualitative study generated five key themes concerning communicating about future uncertainty in advanced multimorbidity. All participants acknowledged the ubiquitous and irreducible nature of uncertainty, but demonstrated different responses to addressing this, comprising acceptance, avoidance and the need for reassurance. While patients’ and informal carers’ experience of communication focused largely on openness and honesty and their relationship with healthcare professionals, healthcare professionals focused more on their levels of clinical experience and their confidence in communicating about future uncertainty.

### What this study adds

We understand that this study is the first to investigate patient, carer and healthcare professional experiences of future uncertainty communication in older adults with advanced multimorbidity. While patients and carers preferred discussion about uncertainties related to managing daily life, healthcare professionals focused more on a narrower concept of prognostic uncertainty, leading to a communication gap with missed opportunities to support patients at a challenging time.^7,28^ However, participants’ reports of shared perspectives showed that a mutually supportive approach to communicating about an uncertain future is possible.^7^

The ways in which healthcare professionals communicate about future uncertainty impacts many aspects of patient experience.^2,6,14,17,43,44^ This was illustrated in our study where patients and carers experienced impersonal communications, or felt rushed and unheard, resulting in reduced satisfaction and additional distress. While openness and honesty are widely recognised as core to healthcare communication,^15,45,46^ our findings emphasise that this also needs to be tailored to patients’ unique experience of advanced multimorbidity and individual priorities.^14,28,44,47^

Although the preference for open and honest communication was prevalent, it was not universal. As noted in other studies,^28,46^ we found variation in information needs and some patients and carers did not want to engage in conversations about the future.^27,48,49^ For these individuals, future uncertainty may be protective, and overtly addressing unknown futures may reduce hope and lead to poorer decision making.^10,25,43,50^ The experience of participants in this study suggests that the onus is on healthcare professionals to initiate dialogue, with a view to shared decision making and informed consent, but also to recognise individual patient preferences shaped by their unique experience of multimorbidity. In some cases, this may mean actively holding uncertainty on behalf of patients, creating what has been termed ‘informed trust’.^51^ Further study is needed to investigate the factors that mediate uncertainty communication preferences, including generational or cultural variations.

While the nature of advanced multimorbidity makes it impossible for healthcare professionals to offer certainty concerning the future, our study suggests an ongoing requirement to provide support for patients even where medical interventions are limited.^34,46,52^ Wellbery notes the value in healthcare professionals seeing medical uncertainty as an opportunity for self-reflection, which will equip them to better address the nuanced experience and different preferences of their patients.^53^ Despite the heterogenous nature of the population with advanced multimorbidity, in the face of an unknown future harnessing future uncertainty in planning for different potential outcomes may help patients and carers move towards a place of ‘safe uncertainty’, by addressing known unknowns within an individual context.^19^

Discussion regarding the need to shift the focus in medicine from definitive diagnoses to acknowledging the unique, complex and nuanced nature of illness and care,^4,6,54^ will be increasingly salient for the growing numbers of older adults with multimorbidity where unpredictable illness trajectories and complex multidisciplinary care are typical.^16,55,56^ Our findings suggest that educational initiatives are needed to enable less experienced healthcare professionals to distinguish between, and communicate, their own uncertainty versus true unknowns.^3^ Common findings such as the importance of open communication and personalised relationships, suggest training should also address techniques to work with patients and carers to formulate future plans within the context of differences in personal responses to uncertainty and experiences of illness.^20,35^ Whilst frameworks and resources exist to support communication of uncertainty in healthcare in general,^57–59^ none focus specifically on the uncertainties engendered by advanced multimorbidity, or on the future.

### Strengths and limitations

This multi-perspective, multi-method, qualitative research obtained a range of patient, informal carer and healthcare professional views and experiences in diverse contexts. Notwithstanding differences in patterns of disease and cultural differences in experiences of uncertainty communication, the common threads of experience across the variety of health conditions in different settings indicate that these findings are also relevant to a wider context. We recruited from settings that enabled us to capture a wide range of perspectives across the spectrum of advanced multimorbidity as intended, however we could not explore experience across all permutations of illness and care context. Therefore, it is not possible to make recommendations for practice from this research that cover every scenario. Despite this limitation, we identified shared experiences across the study population indicating sufficient information power to answer the focused research question. Further research could explore experience in other care contexts such as community or acute care, or in specific diagnostic combinations. The patient sample was not ethnically diverse, and participants only identified three informal carers meaning their perspective was underrepresented. We deliberately included healthcare professional participants who were experienced in uncertainty communication; future research could specifically explore a wider range of healthcare professionals’ experiences, including those for whom uncertainty communication is a less core aspect of their role. Data from primary care and general practice would add another lens on future uncertainty communication.

## Conclusion

Uncertainty about the future is integral to the experience of older adults with advanced multimorbidity. The ways in which this uncertainty is communicated can either support patients and carers to safely engage with it or further add to their distress. Clinical experience is important in enabling healthcare professionals to sensitively communicate uncertainty, therefore additional training for all healthcare professionals to increase their confidence in discussing uncertainty is needed. This should largely focus on open and honest conversations with patients and carers but also be sensitive to addressing each individual’s preferences based on their unique experiences of multimorbidity.

## Supplemental Material

sj-docx-1-pmj-10.1177_02692163251393586 – Supplemental material for ‘Adrift in a sea of just absolute unknowableness’: A multimethod qualitative study exploring patient, carer and healthcare professional experiences of communicating about future uncertainty in multimorbidity Supplemental material, sj-docx-1-pmj-10.1177_02692163251393586 for ‘Adrift in a sea of just absolute unknowableness’: A multimethod qualitative study exploring patient, carer and healthcare professional experiences of communicating about future uncertainty in multimorbidity by Rosanna Fennessy, Anna Spathis, Stephen Barclay and Simon Noah Etkind in Palliative Medicine

sj-docx-2-pmj-10.1177_02692163251393586 – Supplemental material for ‘Adrift in a sea of just absolute unknowableness’: A multimethod qualitative study exploring patient, carer and healthcare professional experiences of communicating about future uncertainty in multimorbidity Supplemental material, sj-docx-2-pmj-10.1177_02692163251393586 for ‘Adrift in a sea of just absolute unknowableness’: A multimethod qualitative study exploring patient, carer and healthcare professional experiences of communicating about future uncertainty in multimorbidity by Rosanna Fennessy, Anna Spathis, Stephen Barclay and Simon Noah Etkind in Palliative Medicine

## References

[1] Han PKJ Klein WMP Arora NK . Varieties of uncertainty in health care: a conceptual taxonomy. Med Decis Making 2011; 31: 828–838. 22067431 10.1177/0272989X11393976 PMC3146626

[2] Etkind SN Li J Louca J , et al Total uncertainty: a systematic review and thematic synthesis of experiences of uncertainty in older people with advanced multimorbidity, their informal carers and health professionals. Age Ageing 2022; 51: 1–11. 10.1093/ageing/afac188 PMC9385183 35977149

[3] Fox RC . The evolution of medical uncertainty. Millbank Meml Fund Quaterly Health Soc 1980; 58(1): 1. 6903782

[4] Simpkin AL Schwartzstein RM . Tolerating uncertainty - the next medical revolution? N Engl J Med 2016; 375: 1713–1715. 27806221 10.1056/NEJMp1606402

[5] Smith AK White DB Arnold RM . Uncertainty–the other side of prognosis. N Engl J Med 2013; 368: 2448–2450. 23802514 10.1056/NEJMp1303295 PMC3760713

[6] Kimbell B Murray SA Macpherson S , et al Embracing inherent uncertainty in advanced illness. BMJ 2016; 354: i3802. 10.1136/bmj.i3802 27430629

[7] Etkind SN Koffman J . Approaches to managing uncertainty in people with life-limiting conditions: role of communication and palliative care. Postgrad Med J 2016; 92: 412–417. 27129911 10.1136/postgradmedj-2015-133371

[8] Nanton V Munday D Dale J , et al The threatened self: considerations of time, place, and uncertainty in advanced illness. Br J Health Psychol 2016; 21: 351–373. 26689299 10.1111/bjhp.12172

[9] Barnett K Mercer SW Norbury M , et al Epidemiology of multimorbidity and implications for health care, research, and medical education: a cross-sectional study. Lancet 2012; 380: 37–43. 22579043 10.1016/S0140-6736(12)60240-2

[10] Mishel MH . Uncertainty in illness. J Nurs Scholarsh 1988; 20: 225–232. 10.1111/j.1547-5069.1988.tb00082.x 3203947

[11] Bowers SP Black P McCheyne L , et al Descriptions of advanced multimorbidity: a scoping review with content analysis. J Multimorb Comorbidity 2025; 15: 26335565251326309. 10.1177/26335565251326309 PMC11920996 40110541

[12] Finucane AM Bone AE Etkind S , et al How many people will need palliative care in Scotland by 2040? A mixed-method study of projected palliative care need and recommendations for service delivery. BMJ Open 2021; 11: e041317. 10.1136/bmjopen-2020-041317 PMC7868264 33536318

[13] Chowdhury SR Chandra Das D Sunna TC , et al Global and regional prevalence of multimorbidity in the adult population in community settings: a systematic review and meta-analysis. med 2023; 57: 101860. 10.1016/j.eclinm.2023.101860 PMC9971315 36864977

[14] Bayliss EA Edwards AE Steiner JF , et al Processes of care desired by elderly patients with multimorbidities. Fam Pract 2008; 25: 287–293. 18628243 10.1093/fampra/cmn040 PMC2504745

[15] Murray SA Boyd K Moine S , et al Using illness trajectories to inform person centred, advance care planning. BMJ 2024; 384: e067896. 10.1136/bmj-2021-067896 38428953

[16] Norman RM Jelin E Bjertnaes O . Multimorbidity and patient experience with general practice: a national cross-sectional survey in Norway. BMC Prim Care 2024; 25: 249. 38987692 10.1186/s12875-024-02495-1 PMC11238367

[17] Mason B Nanton V Epiphaniou E , et al ‘My body’s falling apart.’ Understanding the experiences of patients with advanced multimorbidity to improve care: serial interviews with patients and carers. BMJ Support Palliat Care 2016; 6: 60–65. 10.1136/bmjspcare-2013-000639 25023218

[18] Salisbury C . Multimorbidity: redesigning health care for people who use it. Lancet 2012; 380: 7–9. 22579042 10.1016/S0140-6736(12)60482-6

[19] Mason B . Towards positions of safe uncertainty. Hum Syst Ther Cult Attach 2022; 2: 54–63.

[20] Patel B Gheihman G Katz JT , et al Navigating uncertainty in clinical practice: a structured approach. J Gen Intern Med 2024; 39: 829–836. 38286969 10.1007/s11606-023-08596-4 PMC11043270

[21] Johnson CG Levenkron JC Suchman AL , et al Does physician uncertainty affect patient satisfaction? J Gen Intern Med 1988; 3: 144–149. 3357071 10.1007/BF02596120

[22] Neville A Jordan A Beveridge JK , et al Diagnostic uncertainty in youth with chronic pain and their parents. J Pain 2019; 20: 1080–1090. 30904516 10.1016/j.jpain.2019.03.004

[23] Bontempo AC . Patient attitudes toward clinicians’ communication of diagnostic uncertainty and its impact on patient trust. SSM - Qual Res Health 2023; 3: 100214.

[24] Sharkiya SH . Quality communication can improve patient-centred health outcomes among older patients: a rapid review. BMC Health Serv Res 2023; 23: 886. 37608376 10.1186/s12913-023-09869-8 PMC10464255

[25] Politi MC Clark MA Ombao H , et al Communicating uncertainty can lead to less decision satisfaction: a necessary cost of involving patients in shared decision making? Health Expect 2011; 14: 84–91. 20860780 10.1111/j.1369-7625.2010.00626.x PMC3010418

[26] Epiphaniou E Shipman C Harding R , et al Avoid ‘prognostic paralysis’–just get ahead and plan and co-ordinate care. NPJ Prim Care Respir Med 2014; 24: 14085. 25297513 10.1038/npjpcrm.2014.85 PMC4373499

[27] Smith TA Disler RT Jenkins CR , et al Perspectives on advance care planning among patients recently requiring non-invasive ventilation for acute respiratory failure: a qualitative study using thematic analysis. Palliat Med 2017; 31: 566–574. 28440124 10.1177/0269216316670286

[28] Myers J Selby D . Personalizing prognosis in a patient with serious illness. Can Med Assoc J 2014; 186: 169–170. 24418981 10.1503/cmaj.131415 PMC3928202

[29] Simpkin AL Armstrong KA . Communicating uncertainty: a narrative review and framework for future research. J Gen Intern Med 2019; 34: 2586–2591. 31197729 10.1007/s11606-019-04860-8 PMC6848305

[30] Etkind SN Barclay S Spathis A , et al Uncertainty in serious illness: a national interdisciplinary consensus exercise to identify clinical research priorities. PLoS One 2024; 19: e0289522. 10.1371/journal.pone.0289522 PMC10903860 38422036

[31] Snaith M Conway N Beinema T , et al A multimodal corpus of simulated consultations between a patient and multiple healthcare professionals. Lang Resour Eval 2021; 55: 1077–1092.

[32] Blackmore A Kasfiki EV Purva M . Simulation-based education to improve communication skills: a systematic review and identification of current best practice. BMJ Simul Technol Enhanc Learn 2018; 4: 159–164. 10.1136/bmjstel-2017-000220 PMC8990192 35519010

[33] Cox C Hatfield T Fritz Z . How and why do doctors communicate diagnostic uncertainty: an experimental vignette study. Health Expect 2024; 27: e13957. 10.1111/hex.13957 PMC10774830 38828702

[34] Bhise V Meyer AND Menon S , et al Patient perspectives on how physicians communicate diagnostic uncertainty: an experimental vignette study. Int J Qual Health Care 2018; 30: 2–8. 29329438 10.1093/intqhc/mzx170

[35] Etkind SN Fennessy R Wang J , et al Communicating prognostic uncertainties in advanced multimorbidity: a multimethod qualitative study to co-design practice recommendations. Eur Geriatr Med 2025; 16: 1217–1229. 40411723 10.1007/s41999-025-01228-6 PMC12378655

[36] Tong A Sainsbury P Craig J . Consolidated criteria for reporting qualitative research (COREQ): a 32-item checklist for interviews and focus groups. Int J Qual Health Care 2007; 19: 349–357. 17872937 10.1093/intqhc/mzm042

[37] Staniszewska S Brett J Simera I , et al GRIPP2 reporting checklists: tools to improve reporting of patient and public involvement in research. BMJ 2017; 3: 13. 10.1186/s40900-017-0062-2 PMC5611595 29062538

[38] Murtagh FE Bausewein C Verne J , et al How many people need palliative care? A study developing and comparing methods for population-based estimates. Palliat Med 2014; 28: 49–58. 23695827 10.1177/0269216313489367

[39] Malterud K Siersma VD Guassora AD . Sample size in qualitative interview studies: guided by information power. Qual Health Res 2016; 26: 1753–1760. 26613970 10.1177/1049732315617444

[40] Braun V Clarke V . Using thematic analysis in psychology. Qual Res Psychol 2006; 3: 77–101.

[41] Braun V Clarke V . Reflecting on reflexive thematic analysis. Qual Res Sport Exerc Health 2019; 11: 589–597.

[42] Creswell JW (ed.). Research design : qualitative, quantitative & mixed methods approaches. 5th ed. Sage Publications Ltd, 2018.

[43] Selman L Harding R Beynon T , et al Improving end-of-life care for patients with chronic heart failure: “Let’s hope it’ll get better, when I know in my heart of hearts it won’t. Heart 2007; 93: 963–967. 17309905 10.1136/hrt.2006.106518 PMC1994396

[44] Moody E Martin-Misener R Baxter L , et al Patient perspectives on primary care for multimorbidity: an integrative review. Health Expect 2022; 25: 2614–2627. 36073315 10.1111/hex.13568 PMC9700181

[45] Engel M Kars MC Teunissen SCCM , et al Effective communication in palliative care from the perspectives of patients and relatives: a systematic review. Palliat Support Care 2023; 21: 890–913. 37646464 10.1017/S1478951523001165

[46] van Vliet L Francke A Tomson S , et al When cure is no option: how explicit and hopeful can information be given? A qualitative study in breast cancer. Patient Educ Couns 2013; 90: 315–322. 21555199 10.1016/j.pec.2011.03.021

[47] Carey I Shouls S Bristowe K , et al Improving care for patients whose recovery is uncertain. The AMBER care bundle: design and implementation. BMJ Support Palliat Care 2015; 5: 12–18. 10.1136/bmjspcare-2013-000634 25183712

[48] Almack K Cox K Moghaddam N , et al After you: conversations between patients and healthcare professionals in planning for end of life care. BMC Palliat Care 2012; 11: 15. 22985010 10.1186/1472-684X-11-15 PMC3517317

[49] Bernard C Tan A Slaven M , et al Exploring patient-reported barriers to advance care planning in family practice. BMC Fam Pract 2020; 21: 94. 32450812 10.1186/s12875-020-01167-0 PMC7249389

[50] Balen NS Merluzzi TV . Hope, uncertainty, and control: a theoretical integration in the context of serious illness. Patient Educ Couns 2021; 104: 2622–2627. 34294492 10.1016/j.pec.2021.07.029

[51] Holton R Fritz Z . Taking responsibility for uncertainty. In: Davies B Levy N De Marco G , et al (eds) Responsibility and Healthcare. Oxford University Press, 2022. 38771922

[52] Sep MS van Osch M van Vliet LM , et al The power of clinicians’ affective communication: how reassurance about non-abandonment can reduce patients’ physiological arousal and increase information recall in bad news consultations. An experimental study using analogue patients. Patient Educ Couns 2014; 95: 45–52. 24485947 10.1016/j.pec.2013.12.022

[53] Wellbery C . The value of medical uncertainty? Lancet 2010; 375: 1686–1687. 20480942 10.1016/s0140-6736(10)60725-8

[54] Malterud K Guassora AD Reventlow S , et al Embracing uncertainty to advance diagnosis in general practice. Br J Gen Pract 2017; 67: 244. 28546389 10.3399/bjgp17X690941 PMC5442924

[55] Oishi A Murtagh FE . The challenges of uncertainty and interprofessional collaboration in palliative care for non-cancer patients in the community: a systematic review of views from patients, carers and health-care professionals. Palliat Med 2014; 28: 1081–1098. 24821710 10.1177/0269216314531999 PMC4232314

[56] Meyer AND Giardina TD Khawaja L , et al Patient and clinician experiences of uncertainty in the diagnostic process: current understanding and future directions. Patient Educ Couns 2021; 104: 2606–2615. 34312032 10.1016/j.pec.2021.07.028

[57] Medendorp NM Stiggelbout AM Aalfs CM , et al A scoping review of practice recommendations for clinicians’ communication of uncertainty. Health Expect 2021; 24: 1025–1043. 34101951 10.1111/hex.13255 PMC8369117

[58] Higginson IJ Koffman J Hopkins P , et al Development and evaluation of the feasibility and effects on staff, patients, and families of a new tool, the Psychosocial Assessment and Communication Evaluation (PACE), to improve communication and palliative care in intensive care and during clinical uncertainty. BMC Med 2013; 11: 213. 24083470 10.1186/1741-7015-11-213 PMC3850793

[59] Khazen M Mirica M Carlile N , et al Developing a framework and electronic tool for communicating diagnostic uncertainty in primary care: a qualitative study. JAMA Netw Open 2023; 6: e232218. 10.1001/jamanetworkopen.2023.2218 PMC9999246 36892841

