# Supplementary material for: ‘Adrift in a sea of just absolute unknowableness’: A multimethod qualitative study exploring patient, carer and healthcare professional experiences of communicating about future uncertainty in multimorbidity
Source: Palliat Med. 2025 Dec 1;40(1):84–94. doi: 10.1177/02692163251393586 (PMC12779763; doi:10.1177/02692163251393586)
Supplement: sj-docx-1-pmj-10.1177_02692163251393586 – Supplemental material for ‘Adrift in a sea of just absolute unknowableness’: A multimethod qualitative study exploring patient, carer and healthcare professional experiences of communicating about future uncertainty in multimorbidity [file sj-docx-1-pmj-10.1177_02692163251393586.docx]

**Supplementary file 1 –**

**Inclusion criteria for defining advanced multimorbidity**

*(based on the Murtagh estimate of palliative care need)^1^*

**Participants must have ≥ 2 conditions listed below**

| **Grouping** | **Conditions included** |
| --- | --- |
| **Cancer** | **All malignant neoplasms**  (except for non-metastatic skin cancers, or those being treated with curative intent) |
| **Organ failure** | **Heart disease and heart failure**  (excluding hypertension, Atrial fibrillation, previous myocardial infarction or arrhythmia with full recovery, asymptomatic heart failure (New York Heart Association (NYHA) 1)  **Chronic lower respiratory disease, respiratory failure**  (excluding asthma, mild COPD)  **Renal failure, reno-vascular disease** (excluding CKD 1 – 3)  **Liver disease** (cirrhosis of any cause) |
| **Dementia** | **Vascular dementia, Alzheimer’s disease, other dementias, senility.**  (unless lacking capacity to participate) |
| **Neurological and other** | **Progressive neurological disease**  (Huntington’s disease, Motor neurone disease, Parkinson’s disease, Progressive Supranuclear Palsy, Multiple sclerosis, Multi system atrophy)  **Haemorrhagic, ischaemic and unspecified stroke**  (exclude if minor stroke with complete or near complete functional recovery)  **Long term musculoskeletal conditions e.g. osteoarthritis, osteoporosis** (if limiting daily activity).  **Other serious or life-limiting illness or impairment not included above**  (including clinical syndromes such as frailty) |

^1^ Murtagh FE, Bausewein C, Verne J, Groeneveld EI, Kaloki YE, Higginson IJ. How many people need palliative care? A study developing and comparing methods for population-based estimates. Palliative medicine. 2014;28(1):49-58.
