# Supplementary material for: ‘Adrift in a sea of just absolute unknowableness’: A multimethod qualitative study exploring patient, carer and healthcare professional experiences of communicating about future uncertainty in multimorbidity
Source: Palliat Med. 2025 Dec 1;40(1):84–94. doi: 10.1177/02692163251393586 (PMC12779763; doi:10.1177/02692163251393586)
Supplement: sj-docx-2-pmj-10.1177_02692163251393586 – Supplemental material for ‘Adrift in a sea of just absolute unknowableness’: A multimethod qualitative study exploring patient, carer and healthcare professional experiences of communicating about future uncertainty in multimorbidity [file sj-docx-2-pmj-10.1177_02692163251393586.docx]

**Supplementary file 2 – Interview topic guides**

**Study title: Experiences and communication of future uncertainty in multimorbidity:**

**Topic guide for patient and carer interviews**

Section 1: Experience of uncertainty and uncertainty communication

**Question.** **To start with I’d like to ask [each of] you to tell me about your daily lives, how they are affected by illness, and how you feel about the future**

*Sub questions and prompts [ask patient and carer in turn].*

- What are you uncertain about when you think ahead to the future? Can you give an example?
- What effect does uncertainty have on you?
- Have you had conversations about uncertainty with health professionals? Please tell me about them.
- [If not expressing uncertainty about the future] how have you overcome uncertainties previously?

Section 2: Response to uncertainty

**Question. When you feel uncertain about the future, how do you respond?**

*Sub questions and prompts.*

- When they feel uncertain about their future some people try to avoid discussing it, whereas others wish to discuss their future in detail, is either of these true for you? Could you say more?
- To what extent do you prefer to know what might happen in future with your illness?
- If healthcare professionals are uncertain about your future illness, what would you want them to do?
- [if not uncertain about the future] how would you respond if you did feel uncertain?

Section 3: Communicating uncertainty

**Question. What is the best way to discuss uncertainty about your future with healthcare professionals?**

*Sub questions and prompts.*

- Who should raise uncertainty first? What would help you to ask about it if you wished to?
- What affects how easy or difficult it is to discuss uncertainty?
- How much information should be shared? What affects this?
- What words or phrases are helpful when discussing uncertainty?
- What might a toolkit to support uncertainty communication look like?

Is there anything else that would improve conversations about uncertainty?

**Study title: Experiences and communication of future uncertainty in multimorbidity:**

**Topic guide for healthcare professional focus groups.**

Section 1: Experience of uncertainty

**Main question.** **To start with I’d like to ask [each of] you to introduce yourself and describe your experience of managing future unknowns when caring for older people with multimorbidity**

*Sub questions and prompts*

- Think of an older patient you have looked after who had multiple advanced illnesses. When caring for that patient, or others like them, what uncertainties were prominent?
- What effects do you think uncertainty has on your patients and their carers?
- What effect does uncertainty have on you and your practice? What challenges does it bring?

Section 2: Response to uncertainty:

**Main question. When you recognise future uncertainty, how do you respond?**

*Sub questions and prompts.*

- How do you respond when you recognise future uncertainty?
  - Do you always share uncertainty?
- How do you respond when your patient/carer raises uncertainty?
- What responses to uncertainty do you think are most helpful/harmful?

Section 3: Communicating uncertainty

**Main question. What approaches do you take when communicating future uncertainty in multimorbidity?**

*Sub questions and prompts.*

- How can future uncertainty best be communicated?
  - What works well in such conversations? What are your top tips?
  - What steps do you take within the conversation? How do you structure the conversation?
  - What are facilitators or barriers to holding such conversations?
- Do you tailor the conversation to patients, and if so how?
  - If patients accept/avoid/address uncertainty, how does this change your communication?
- How does the presence of multimorbidity affect conversations about future uncertainty?
- What would change how you communicate about uncertainty or the information you share?
- What would a toolkit to aid communication of uncertainty look like?
